# Supplementary material for: Orexin modulates behavioral fear expression through the locus coeruleus
Source: Nat Commun. 2017 Nov 20;8:1606. doi: 10.1038/s41467-017-01782-z (PMC5694764; doi:10.1038/s41467-017-01782-z)
Supplement: Supplementary file 1 — Supplementary information [file 41467_2017_1782_MOESM1_ESM.pdf]

Supplementary information

Supplementary figures

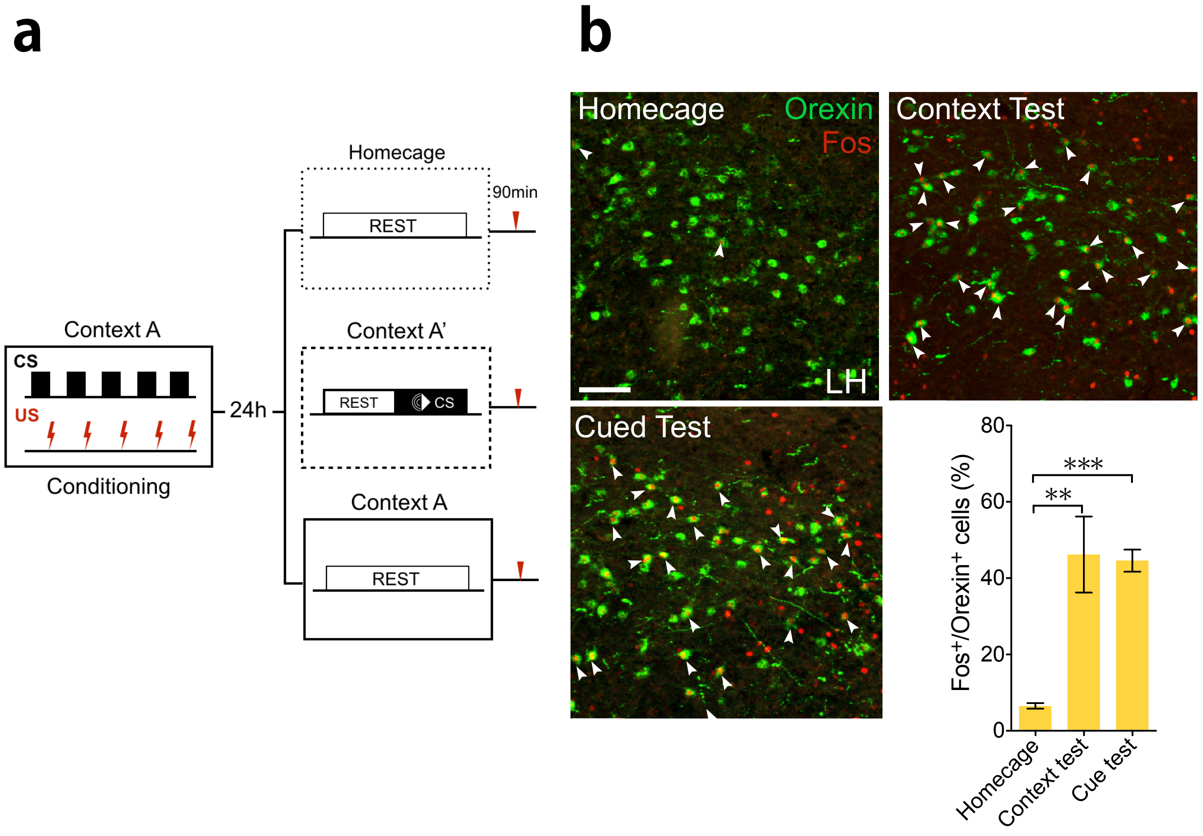

Supplementary Fig. 1 | Orexin neurons are activated by emotionally salient cues and contexts. (a) Experimental protocol. (b) Images show Fos immunoreactivity in orexin neurons in cued (n = 4) and contextual (n = 3) fear test compared to home cage control (n = 3) at 24 h after conditioning (Homecage, n = 4; Context test, n = 3; Cued test, n = 4: One-Way ANOVA with Sidak's post-hoc test, $F_{(2, 8)} = 21.81$, $p = 0.0006$). Values are presented as mean ± SEM. $^{**}p < 0.01$, $^{***}p < 0.001$. Scale bar, 100 µm.

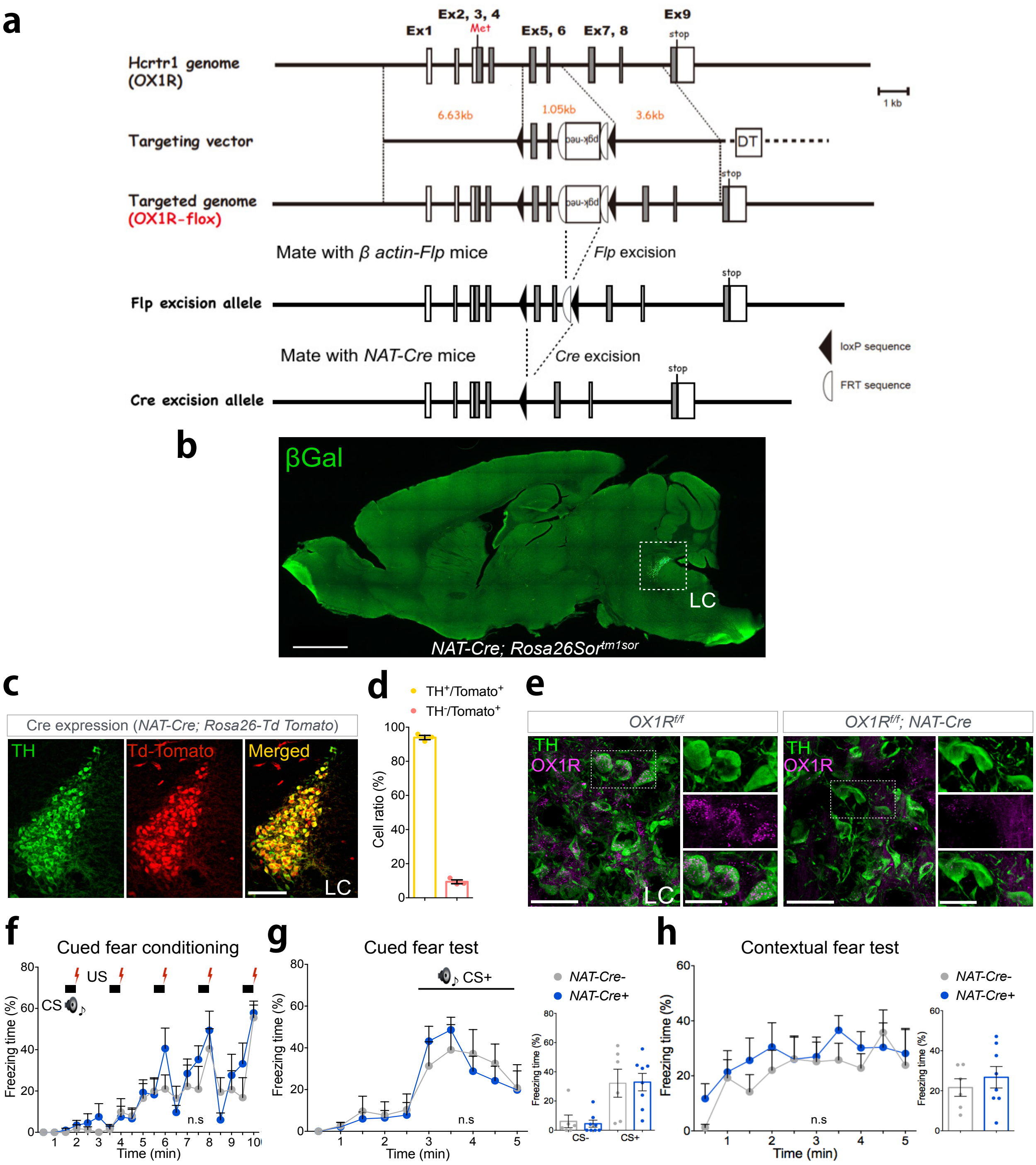

Supplementary Fig. 2 | Generation and analysis of *OX1R* floxed mouse. (a) Schematic representation of strategy to generate *OX1R*$^{f/f}$ mice. Mice containing targeted allele were mated with *Flp* mice to remove the *Neo* cassette. Then, these mice were crossed with *NAT-Cre* mice to excise Exons 5 and 6 especially in NA^{LC} neurons. (b) Specific Cre expression in NA^{LC} neurons in *NAT-Cre* mated with *Rosa26Sor*^{tm1sor} mice stained with β-galactosidase antibody. Scale bar, 2 mm. (c) Cre-mediated recombination in NA^{LC} neurons of *NAT-Cre* mice mated with *Rosa26-Td Tomato* reporter mice is visualized by red fluorescence. Scale

bar, 100 µm. (d) Most of the TH-positive neurons in LC were Td-Tomato positive (96.01 ± 0.7%, n = 3) and there were a few population of the TH-negative and Td-Tomato positive cells around LC (9.39 ± 1.1%, n = 3). (e) We confirmed specific deletion of *OX1R* mRNA in NA^{LC} neurons with combination of *in situ* hybridization and immunohistochemistry for detecting *OX1R* and TH. Scale bars, 50 µm (Left, low magnified), 25 µm (Right, high magnified). (f-h) *NAT-Cre* mice showed normal freezing response in cued fear conditioning (*NAT-Cre-*; n = 6, *NAT-Cre+*; n = 8: Two-Way RM ANOVA, Sidak's post-hoc test, $F_{(1, 12)}$ = 0.4461, p = 0.5168), cued fear test (Freezing overtime: Two-Way RM ANOVA, Sidak's post-hoc test, $F_{(1, 12)}$ = 0.0036, p = 0.9529, left; Average freezing: Average freezing: unpaired two-tailed Student's t-test, t = 0.0667, p = 0.9483, right) and contextual fear test (Freezing overtime: Two-Way RM ANOVA, Sidak's post-hoc test, $F_{(1, 12)}$ = 0.4973, p = 0.4941, left; Average freezing: Average freezing: unpaired two-tailed Student's t-test, t = 0.7420, p = 0.4724, right) . Values are presented as mean ± SEM.

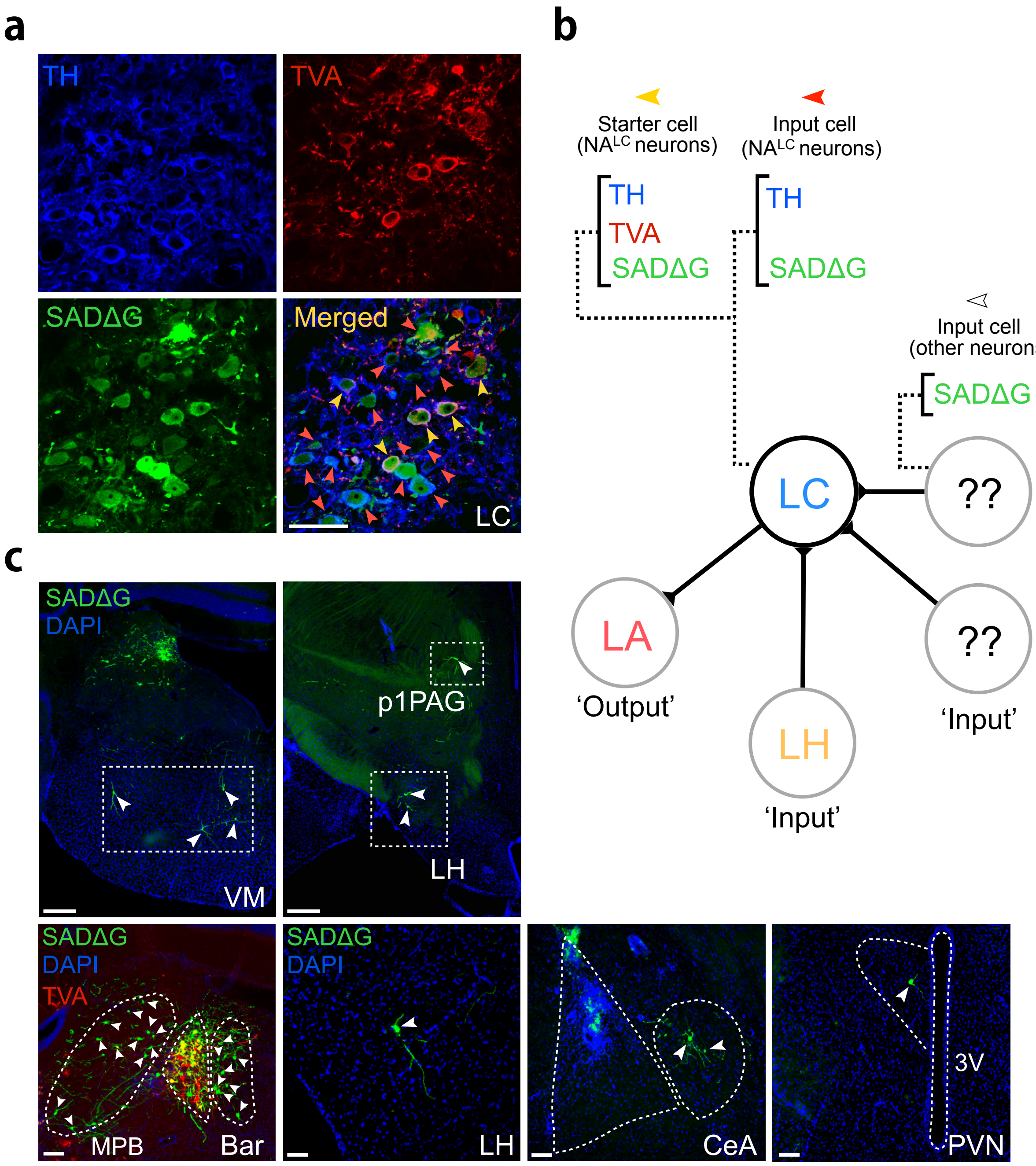

Supplementary Fig. 3 | Input of NA$^{LC\rightarrow LA}$ neurons revealed by cTRIO method. (a) Images show TH^{+}/TVA^{+}/SADΔG^{+} cells (starter cells: yellow arrow heads) and TH^{+}/SADΔG^{+} cells [input cells (NA^{LC}): red arrow heads]. Scale bar, 50 µm. (b) Cartoon showing classification of starter cells (NA^{LC} neurons), input cells (NA^{LC} neurons) and input cells of other neurons (SADΔG^{+} cells; white arrow heads). (c) Images showing input cells in the ventral medulla (VM), medial parabrachial nucleus (MPB), Barrington's nucleus (Bar), periaqueductal gray (p1PAG), lateral hypothalamus (LH), central amygdala (CeA), and Paraventricular nucleus (PVN). Scale bars, 300 µm (upper), 100 µm (lower).

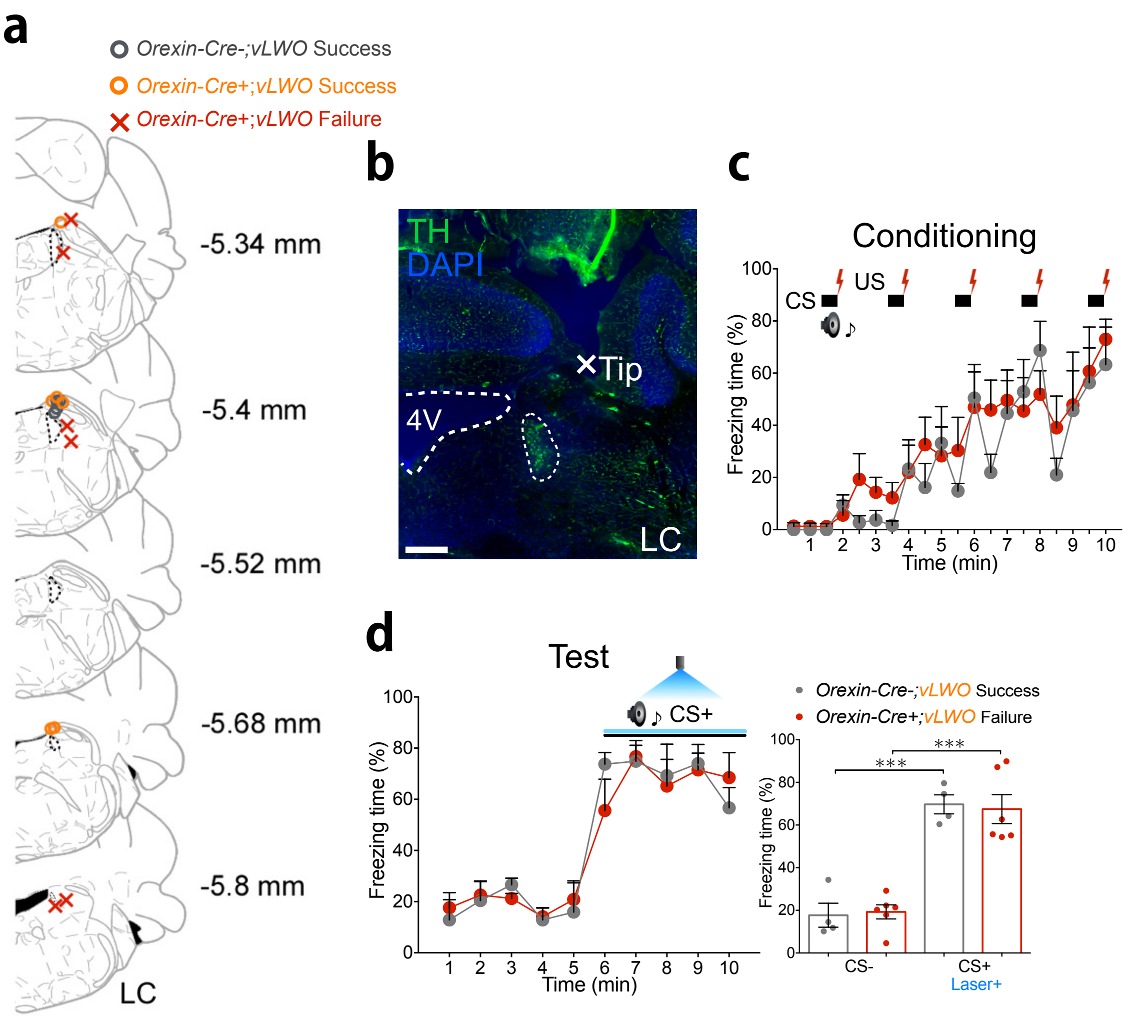

Supplementary Fig. 4 | Trials with failed optic fiber placement in vLWO-mediated inhibition showed no effect on freezing behavior. (a) Positions of optical fiber tip placement. We excluded the trial if the tip was not directly above the LC or the tip itself severely destroyed LC. (b) Representative image showing failed tip placement stained with TH and DAPI. (c) Results of fear conditioning with *Orexin-Cre-;vLWO* Success and *Orexin-Cre+;vLWO* Failure group, showing no difference between these groups (*Orexin-Cre-;vLWO* Success, $n = 4$; *Orexin-Cre+;vLWO* Failure, $n = 6$: Two-Way RM ANOVA, Sidak's post-hoc test, $F_{(1, 8)} = 0.2584$, $p = 0.6249$). (d) The failed tip placement group showed significantly longer freezing time similar to the *Orexin-Cre-;vLWO* Success group (Freezing overtime: Two-Way RM ANOVA with Sidak's post-hoc test, $F_{(1, 8)} = 0.0029$, $p = 0.9582$, left; Average freeizing: unpaired two-tailed Student's *t*-test, $t = 0.2748$, $p = 0.7906$, right). Values are presented as mean ± SEM. ***p <0.001. Scale bar, 300 µm.

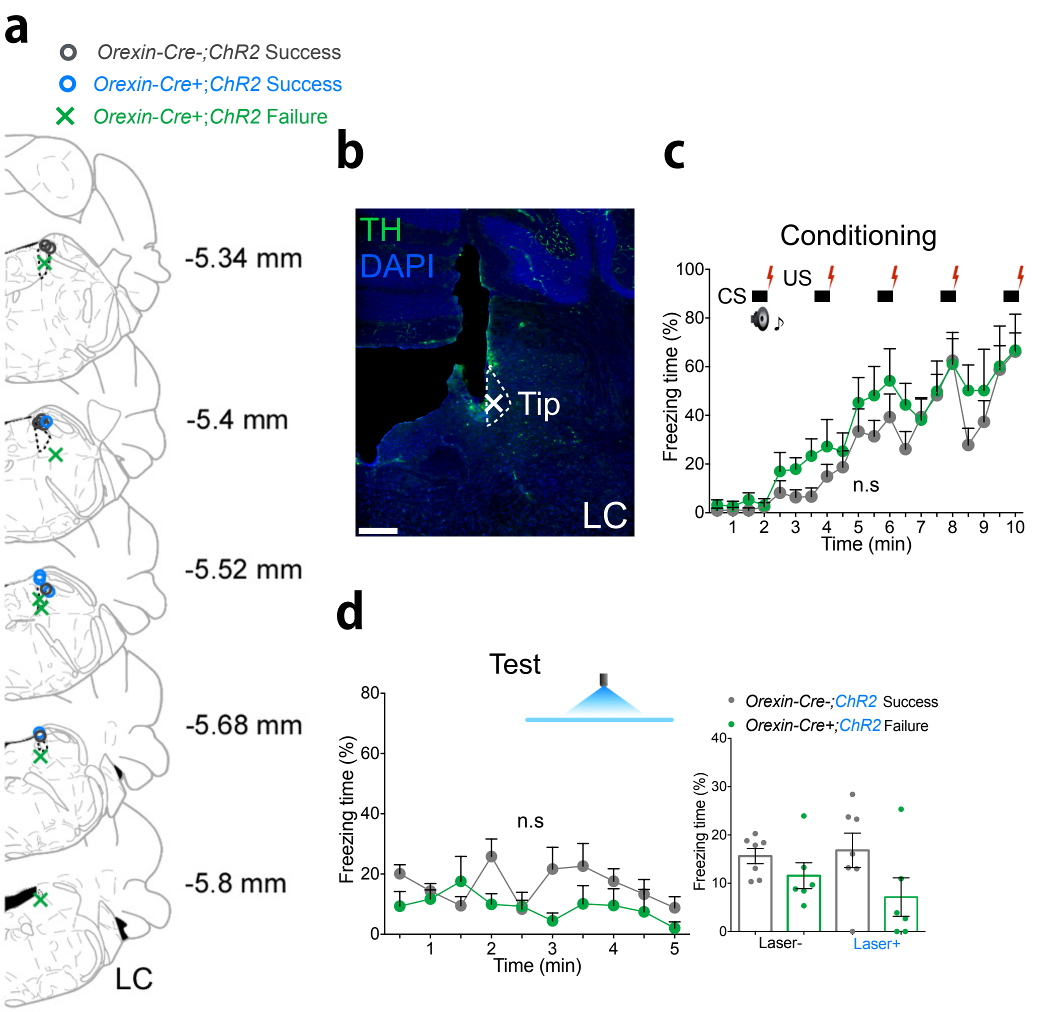

Supplementary Fig. 5 | ChR2-mediated stimulation of orexin fibers showed no effect on freezing behavior. (a) Place of optical fiber tip at the LC for laser stimulation of orexin$^{LH\rightarrow LC}$ confirmed after cued fear conditioning. We excluded the trial if the tip was not precisely placed above the LC or the tip itself severely destroyed the LC. (b) Representative image showing failed tip placement stained with TH and DAPI. (c) Results of fear conditioning with *Orexin-Cre-;ChR2* Success and *Orexin-Cre+;ChR2* Failure group showing no difference between these groups (*Orexin-Cre-;ChR2* Success, $n = 7$; *Orexin-Cre+;ChR2* Failure, $n = 6$: Two-Way RM ANOVA, Sidak's post-hoc test, $F_{(1, 11)} = 1.104$, $p = 0.3160$). (d) The *Orexin-Cre+;ChR2* Failure group with laser stimulation didn't show any increase in freezing time similar to the *Orexin-Cre-;ChR2* Success group (Freezing over time: Two-Way RM ANOVA with Sidak's post-hoc test, $F_{(1, 11)} = 3.191$, $p = 0.1016$, left; Average freezing: unpaired two-tailed Student's t-test, $t = 1.808$, $p = 0.0992$, right). Values are presented as mean ± SEM. Scale bar, 300 µm.

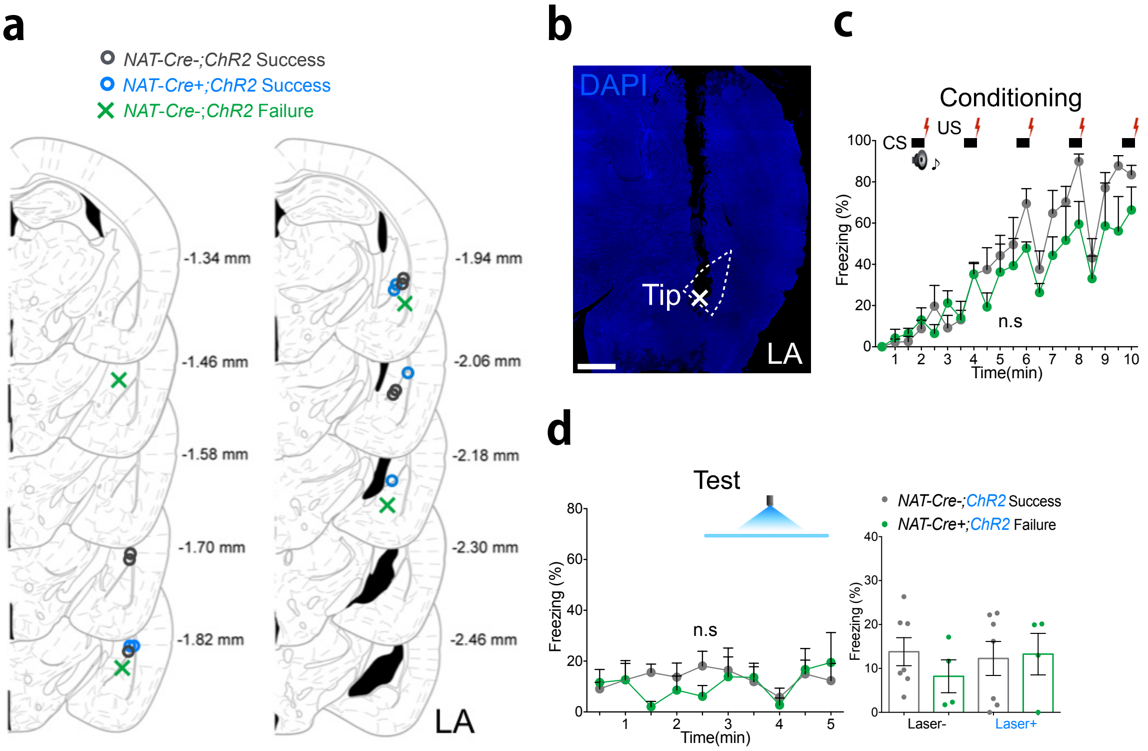

Supplementary Fig. 6 | Trials with failed optic fiber placement showed no effect on freezing behavior. (a) Summary of optical fiber tip placements for laser stimulation of $NA^{LC \rightarrow LA}$ after cued fear conditioning. We excluded the trial if the tip was not precisely above the LA or the tip itself severely destroyed the LA. (b) Representative image showing failed tip placement around the LA stained with DAPI. (c) Results of cued fear conditioning with *NAT-Cre-;ChR2* Success and *NAT-Cre+;ChR2* Failure groups showing no difference between these groups (*NAT-Cre-;ChR2* Success, $n = 7$; *NAT-Cre+;ChR2* Failure, $n = 4$: Two-Way RM ANOVA, Sidak's post-hoc test, $F_{(1, 9)} = 2.406$, $p = 0.1553$). (d) The *NAT-Cre+;ChR2* Failure group didn't show any increase in freezing time with laser stimulation similar to the *NAT-Cre-;ChR2* Success group (Freezing over time: Two-Way RM ANOVA, Sidak's post-hoc test, $F_{(1, 9)} = 0.1852$, $p = 0.6770$, left; Average freezing: unpaired two-tailed Student's *t*-test, $t = 0.1635$, $p = 0.8749$, right). Values are presented as mean ± SEM. Scale bar, 600 μm.

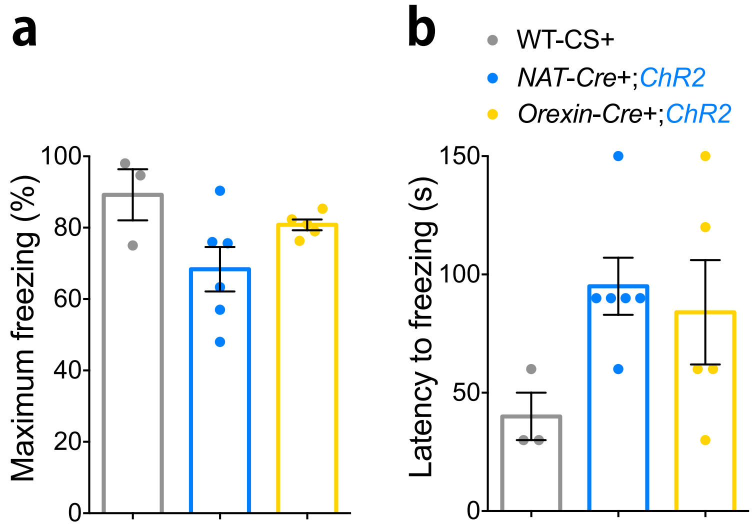

Supplementary Fig. 7 | Optogenetic stimulation of NA^{LC} or orexin^{LH} fibers induced increased but delayed freezing behavior. (a) There was no difference in maximum freezing time between ones induced by150 s CS presentation or laser stimulation (WT-CS, n = 3; *NAT-Cre+;ChR2*, n = 6; *Orexin-Cre+;ChR2*, n = 4: One-Way ANOVA with Tukey's post hoc test, $F_{(2, 11)} = 3.492$, $p = 0.0669$). (b) Photostimulation of NA^{LC} neurons or orexin^{LH} fibers showed a tendency to induce a delayed freezing response compared with that induced by auditory CS after cued fear conditioning (WT-CS, n = 3; *NAT-Cre+:ChR2*, n = 6; *Orexin-Cre+:ChR2*, n = 5: One-Way ANOVA with Tukey's post hoc test, $F_{(2, 11)} = 2.333$, $p = 0.1430$). Values are presented as mean ± SEM.

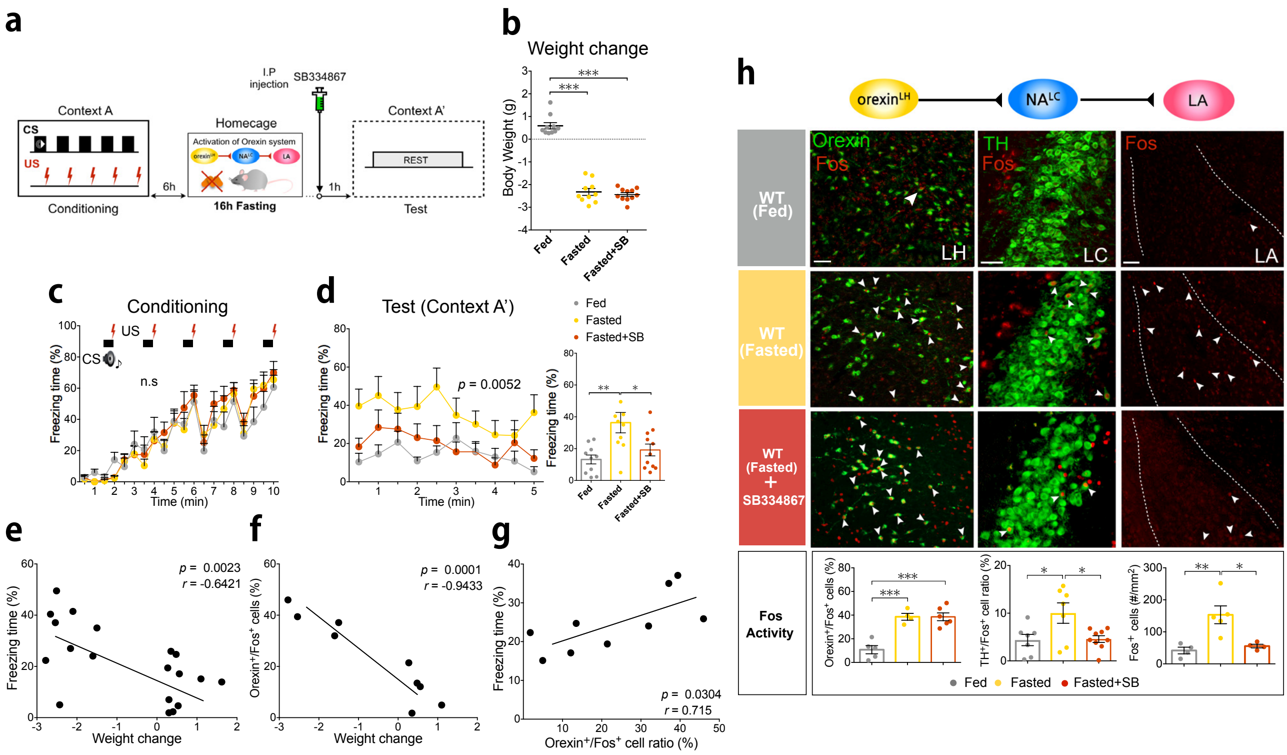

Supplementary Fig. 8 | Fasting increases freezing which is dependent on OX1R. (a) Schematic representation of strategy of fasting manipulation (b) 16 h fasting induced significant reduction of mouse's body weight (Fed, n = 10; Fasted, n = 10; Fasted+ SB, n = 11: One-Way ANOVA with Sidak's post-hoc test, $F_{(2, 28)} = 185.1$, $p < 0.0001$). (c) Result of cued fear conditioning before fasting (Two-Way RM ANOVA with Tukey's post-hoc test, $F_{(2, 28)} = 0.2796$, $p = 0.7582$). (d) Fasted mice showed increased freezing time even in context A' as compared to Fed mice. Fasted mice treated with SB334867(SB) showed almost the same tendency to Fed mice (Two-Way RM ANOVA with Tukey's post hoc test, $F_{(2, 28)} = 6.374$, $p = 0.0052$). (e) Fasting-induced freezing was negatively correlated with the weight change (n = 20, r = -6.421, p = 0.0023). (f) Activation of orexin^{LH} neurons was negatively correlated with the weight change (n = 9, r = -0.9433, p = 0.0001). (g) Fasting-induced freezing was positively correlated with the activation of orexin^{LH} neurons (n = 9, r = 0.715, p = 0.0304). (h) orexin^{LH} neurons, NA^{LC} neurons and LA neurons were highly activated by fasting condition, and intraperitoneal injection of SB334867 inhibited the activation of NA^{LC} and downstream LA neurons (orexin^{LH}; Fed, n = 5; Fasted, n = 4; Fasted+SB, n = 6: One-Way ANOVA with Sidak's post-hoc test, $F_{(2, 12)} = 23.28$, $p < 0.0001$; NA^{LC}; Fed, n = 7; Fasted, n = 7; Fasted+SB, n = 9: One-Way ANOVA with Tukey's post hoc test, $F_{(2, 20)} =$ 5.202, $p = 0.0152$; LA; Fed, n = 4; Fasted, n = 5; Fasted+SB, n = 4: One-Way ANOVA with

Tukey's post hoc test, $F_{(2, 10)} = 9.967$, $p = 0.0042$). Images show the Orexin^{+}/Fos^{+} cells in orexin neurons (left column), TH^{+}/Fos^{+} cells in NA^{LC} neurons (center column), Fos^{+} cells in the LA region (mm^{2}) (right column) in each group. Scale bars: 100 µm. Bottom figures show the percentage of these cells in different regions regarding Fed, Fasted, Fasted+SB groups. Values are presented as mean ± SEM. * p <0.05, ** $p < 0.01$, *** $p < 0.001$. Scale bar, 500 µm.

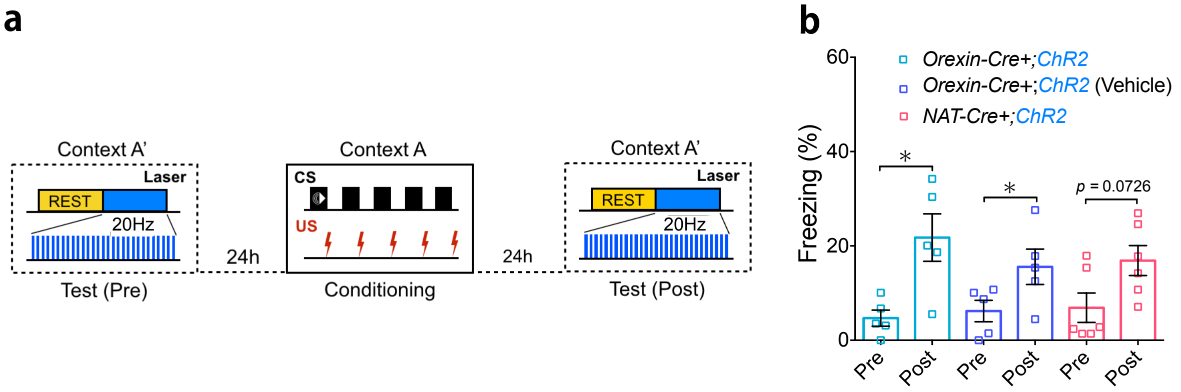

Supplementary Fig. 9 | Change of baseline freezing after fear conditioning. (a) Schematic drawing showing experimental protocol. (b) Baseline freezing time significantly changed because of the fear conditioning manipulation (*Orexin-Cre+;ChR2*, $n = 5$; *Orexin-Cre+;ChR2* (Vehicle), $n = 5$; *NAT-Cre+;ChR2*, $n = 6$: Pre vs Post, unpaired two-tailed Student's t-test, $t = 3.216$, $p = 0.0241$; $t = 2.247$, $p = 0.0484$; $t = 2.136$, $p = 0.0726$). Values are presented as mean ± SEM. *$p < 0.05$.
