# Supplementary material for: Orexin modulates behavioral fear expression through the locus coeruleus
Source: Nat Commun. 2017 Nov 20;8:1606. doi: 10.1038/s41467-017-01782-z (PMC5694764; doi:10.1038/s41467-017-01782-z)
Supplement: Supplementary file 3 — Description of Additional Supplementary Information [file 41467_2017_1782_MOESM3_ESM.pdf]

Description of Additional Supplementary Files

File Name: Supplementary Movie 1
Description: Representative behavior of *Orexin-Cre+AAV-ChR2* mice with 150 s laser stimulation in LC. The first 30 s shows behavior with no light, and the subsequent 150 s period shows behavior with laser stimulation. This movie is played at x4 normal speed. Freezing response markedly increases during the period of photostimulation.

File Name: Supplementary Movie 2
Description: Representative behavior of *NAT-Cre+AAV-ChR2* mice with 150 s laser stimulation. The first 30 s shows behavior with no light, and the subsequent 150 s period shows behavior with laser stimulation. This movie is played at x4 normal speed. Freezing response also increases during the period of photostimulation.

File Name: Supplementary Movie 3
Description: Control mouse (WT+*AAV-ChR2*) with 150 s laser stimulation of NA^{LC}→LA pathway. The first 30 s of the movie shows rest period with no light, and the subsequent 150 s period with shows laser stimulation. This movie is played at x4 normal speed. The control mouse doesn’t show any behavioral change with photostimulation.
